# An E3 ligase TRIM1 promotes colorectal cancer progression via K63-linked ubiquitination and activation of HIF1α

**DOI:** 10.1038/s41389-024-00517-2

**Published:** 2024-05-20

**Authors:** Liuliu Shi, Xianglan Fang, Lijie Du, Jin Yang, Juan Xue, Xiaokai Yue, Duoshuang Xie, Yuanjian Hui, Kun Meng

**Affiliations:** 1 Institute of Infection and Immunity, Department of Infection Control, School of Public Health, Affiliated Taihe Hospital, Hubei University of Medicine, Shiyan, Hubei China; 2 Hubei Key Laboratory of Embryonic Stem Cell Research, School of Basic Medicine, Hubei University of Medicine, Shiyan, China; 3 Hubei Provincial Clinical Research Center for Umbilical Cord Blood Hematopoietic Stem Cells, Taihe Hospital, Hubei University of Medicine, Shiyan, Hubei China; 4 Department of General Surgery, Affiliated Taihe Hospital, Hubei University of Medicine, Shiyan, Hubei China

**Keywords:** Ubiquitylation, Innate immunity, Tumour biomarkers

## Abstract

Accumulating studies have shown that E3 ligases play crucial roles in regulating cellular biological processes and signaling pathways during carcinogenesis via ubiquitination. Tripartite-motif (TRIM) ubiquitin E3 ligases consist of over 70 members. However, the clinical significance and their contributions to tumorigenesis remain largely unknown. In this study, we analyzed the RNA-sequencing expression of TRIM E3 ligases in colorectal cancer (CRC) and identified 10 differentially expressed genes, among which TRIM1 expression predicted poor prognosis of CRC patients. We demonstrated that TRIM1 expression is positively associated with CRC pathological stages, and higher expression is positively correlated with infiltrating levels of immune cells and immunotherapy biomarkers. TRIM1 expression promotes the proliferation and migration of colorectal cancer cells in vitro and in vivo. Transcriptional analysis showed that TRIM1 is responsible for metabolism promotion and immune suppression. Mechanistically, we found that TRIM1 binds HIF1α and mediates its K63-linked ubiquitination, which is required for HIF1α nuclear translocation and subsequent activation. Ubiquitination occurs at Lys214 in the loop between the two PAS domains of HIF1α, and mutation of Lys214 severely disturbs the function of HIF1α. Besides, HIF1α ubiquitination enhances its binding with proteins involved in cellular trafficking and nucleocytoplasmic transport pathway. Collectively, our results indicate TRIM1’s role in predicting prognosis and reveal how TRIM1 functions to upregulate HIF1α expression and promote tumor cell proliferation.

## Introduction

Colorectal cancer is becoming the predominant cancer and the second leading cause of death in cancer patients [1, 2]. It is estimated that about 1.9 million new cases of colorectal cancer worldwide in 2020, of which over 930,000 cases died [3]. Although significant advances in clinical diagnosis, anti-tumor drug discovery and anticancer therapeutics have been achieved, the prognosis remains unoptimistic for lack of the exact molecular diagnosis of CRC. Thus, it is urgent and essential to investigate the molecular mechanisms underlying cancer tumorigenesis and progression, which may be of great significance in developing novel and efficient biomarkers.

Ubiquitination is a highly conserved biological process across eukaryotic organisms and is important for regulating basic cellular processes such as cell cycle, immune invasion, and protein degradation [4]. Three classes of enzymes are involved in this process: the Ub-activating enzyme (E1), the Ub-conjugating enzyme (E2), and the Ub-ligase (E3) [5]. There is increasing evidence that E3 ligase plays a critical role in controlling the development of cancers and is becoming an attractive target for cancer therapies [6, 7]. For example, MDM2 promotes carcinogenesis and metastasis by targeting p53 for proteasomal degradation [8]. SCF^FBXW7^ functions as a tumor suppressor by inhibiting cell cycle progression [9]. The tripartite-motif (TRIM) ubiquitin ligases are a large family of E3 ligases with over 70 members [10]. However, these proteins’ clinical significance and biological functions in cancer remain largely unknown.

In this study, we first identified the differentially expressed TRIM in CRC and their associations with prognosis in CRC. We found TRIM1 was downregulated, and overexpression in CRC was positively associated with poor prognosis and immunotherapy biomarkers. TRIM1 overexpression promotes the proliferation of CRC cells, facilitates metabolism, and restrains immune response. Mechanistically, TRIM1 interacts with and catalyzes K63-linked ubiquitination of HIF1α at Lys 214, which is required for HIF1α nuclear translocation and subsequent activation. Mutation of Lys214 to Arg severely decreased HIF1α’s activity and nuclear localization in CRC cells. In addition, HIF1α ubiquitination enhances its associations with proteins involved in the nucleocytoplasmic transport pathway. Our data highlights TRIM1’s role in predicting prognosis in CRC and reveals its unprecedented functions in regulating tumor cell proliferation.

## Results

### Landscape of expression pattern of TRIM E3 ligases in human CRC sample

**Fig. 1:**
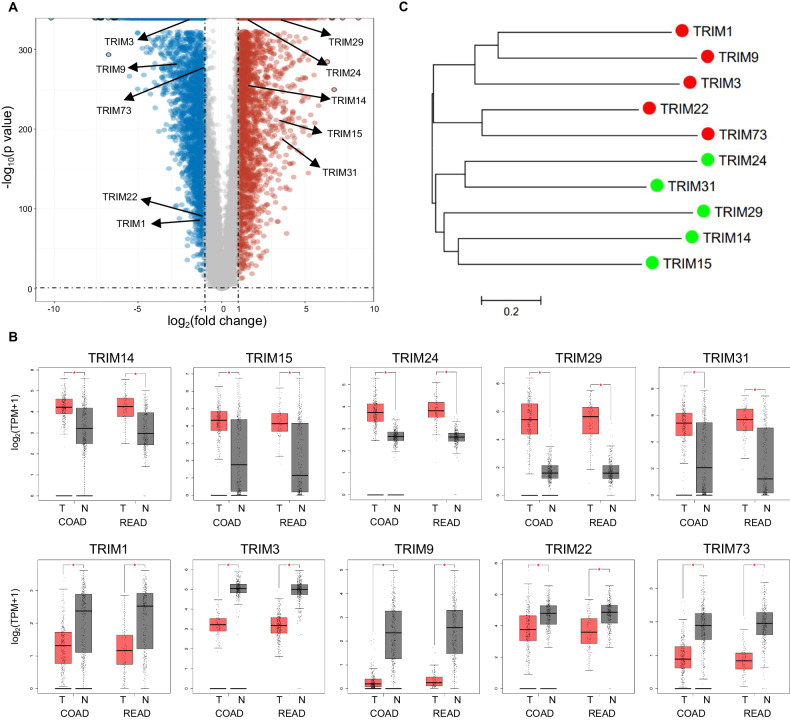
Identification of differentially expressed TRIMs in colorectal cancer. **A** Volcano map showing the overall transcriptional expression in CRC of tumor tissues (*n* = 620) matching the TCGA data and normal tissues (*n* = 830) matching the TCGA normal and GTEx data. Red dots refer to significantly up-regulated genes, blue dots correspond to the down-regulated genes, and gray dots indicate the non-significant change in gene expression. **B** Box plot showing the mRNA expression of differentially expressed TRIMs in COAD (*n* = 275 for tumor tissue and *n* = 349 for normal tissue) and READ (*n* = 92 for tumor tissue and *n* = 318 for normal tissue) from the TCGA normal and GTEx data. **C** Phylogenetic analyses of differentially expressed TRIMs. The amino acid sequence of TRIMs was aligned, and a phylogenetic tree was constructed in MEGA 5.0 using the neighbor-joining method.

To identify the differentially expressed genes in CRC, we analyzed the RNA-sequencing expression profiles for CRC downloaded from the TCGA dataset (https://portal.gdc.com). A volcano plot showed that 10 TRIMs in CRC samples were differentially expressed, while other TRIMs were unchanged (Fig. 1A). Five TRIM expression was significantly upregulated (TRIM14, TRIM15, TRIM24, TRIM29, and TRIM31), and the other five TRIM genes showed decreased expression (TRIM1, TRIM3, TRIM9, TRIM22, and TRIM73) in both colon cancer (COAD) and rectal cancer (READ) (Fig. 1A, B). Interestingly, the downregulated TRIMs and the upregulated TRIMs formed two phylogenetically distinct clusters, indicating their synergistic and divergent roles in CRC cancer development (Fig. 1C).

### Higher expression of TRIM1 predicts poor prognosis in CRC

**Fig. 2:**
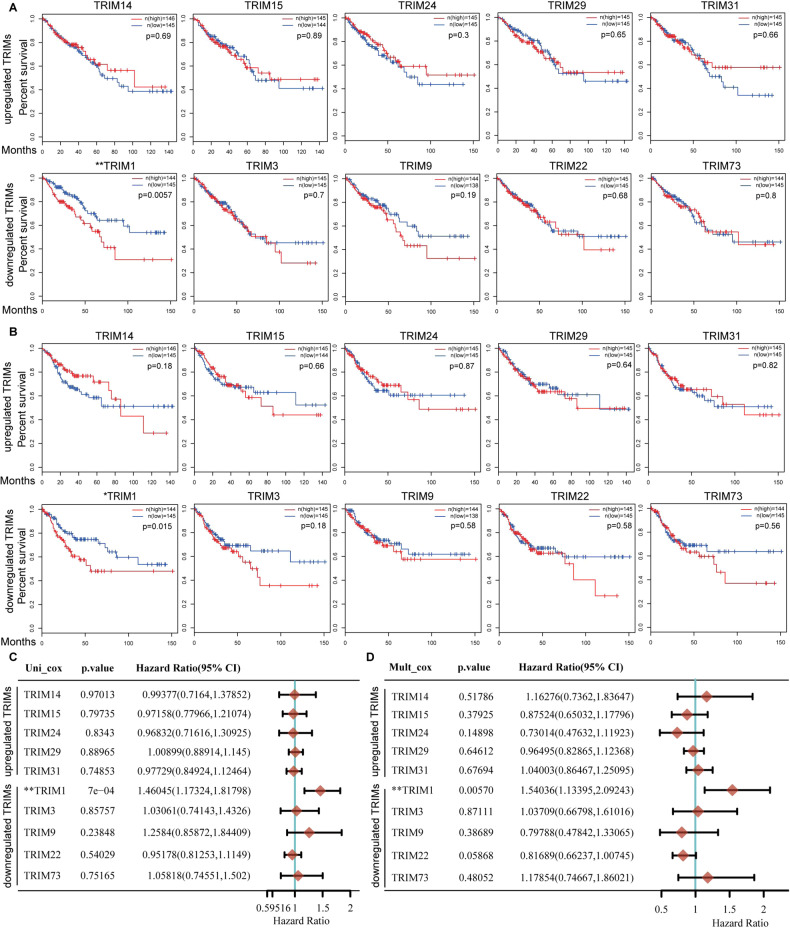
Correlation between TRIMs expression and survival rate of CRC patients. **A**, **B** Kaplan–Meier plots for the survival of CRC patients stratified by the mRNA expression level of each differentially expressed TRIMs. The overall survival curves are shown in (**A**). The disease-free survival curves are shown in (**B**). **C**, **D** Cox regression analysis of mRNA expression of each differentially expressed TRIMs in CRC patients from TCGA data (*n* = 620). The *p* value, hazard ratio (HR), and confidence interval of each TRIM in CRC are analyzed by univariate (**C**) and multivariate (**D**) Cox regression analysis. *p < 0.05, **p < 0.01.

To determine the significance of these differentially expressed TRIMs in CRC, we analyzed their associations with the prognostic value of CRC patients. We used the RNA-sequencing expression profiles and corresponding clinical information for CRC from the TCGA dataset. Kaplan–Meier survival curve showed that a high mRNA level of TRIM1 was significantly associated with poor overall survival (OS) and disease-free survival (DFS) in CRC (Fig. 2A, B). Univariate and multivariate Cox regression analyses exhibited that TRIM1 was an independent prognostic factor (Fig. 2C, D). However, the associations of other TRIMs with survival rates and prognostic values in CRC were not significant. These results showed that only TRIM1 expression could predict prognosis in CRC, emphasizing its role in CRC tumorigenesis. Hence, we choose TRIM1 for the subsequent investigations.

### TRIM1 expression is downregulated in CRC

**Fig. 3:**
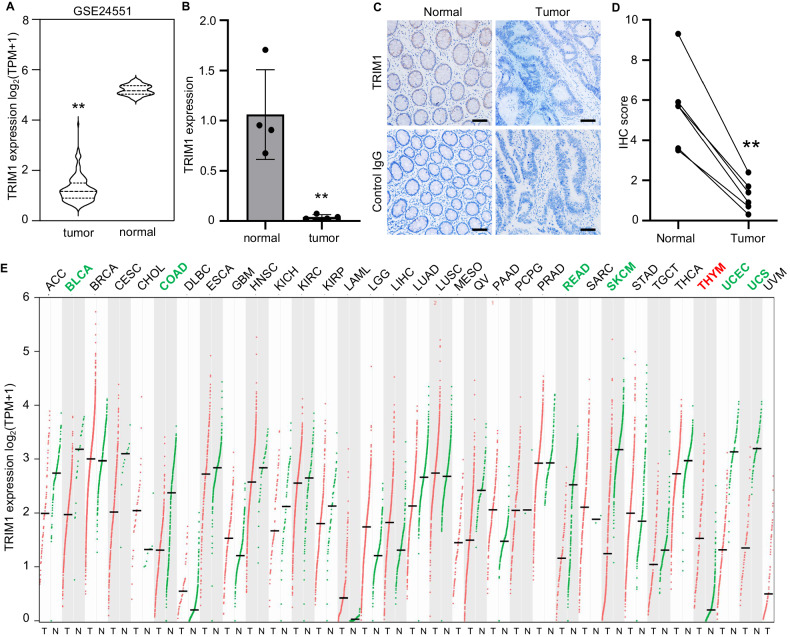
TRIM1 expression is significantly downregulated in colorectal cancer. **A** The mRNA expression of TRIM1 of CRC tumor tissues and their corresponding adjacent normal tissues matching GSE24551 data (*n* = 160 for tumor tissue and *n* = 13 for normal tissue). **B** The mRNA expression of TRIM1 in the tumor tissues compared to the adjacent normal tissues from four CRC patients paired samples. **C**, **D** Immunohistochemical staining of TRIM1 protein in paired samples from six CRC patients. Representative IHC images of TRIM1 were shown (**C**), and the IHC scores were calculated (**D**). **E** The transcriptional expression profile of TRIM1 in 33 types of tumor tissues (T) in TCGA and normal tissues (N) matching the TCGA normal and GTEx data. Red and green labels correspond to the cancer types in which TRIM1 expression is up- and down-regulated in tumor tissue. Scale bar, 50 μm. **p < 0.01.

The expression of TRIM1 was observed to be downregulated in the TCGA dataset (Fig. 1). To further verify this, we have provided another three pieces of evidence. An independent CRC cohort (GSE244551) containing normal and cancer tissues was examined. The results showed that TRIM1 expression was significantly upregulated in the normal tissues (Fig. 3A). Next, we collected four pairs of clinical samples containing the cancer and their adjacent tissues and found that TRIM1 was also upregulated expressed in the adjacent normal tissues (Fig. 3B). In addition, we evaluated the protein expression of TRIM1 in cancer tissues and the adjacent normal tissues of the colon using immunohistochemistry. The results showed that TRIM1 protein was mainly located around the glandular structure of the lumen and was relatively lowly expressed in CRC tissue (Fig. 3C, D). Besides, we examined the TRIM1 expression profile of TRIM1 among different cancers in the TGCA cohort using the GEPIA web tool. Compared to the normal tissues, TRIM1 is downregulated in the six types of cancer, including BLCA, COAD, READ, SKCM, UCEC, and UCS, while upregulated in THYM cancer, suggesting the expression varies among different cancers (Fig. 3E).

### TRIM1 is positively correlated with clinicopathological parameters and immunotherapy biomarkers of CRC

**Fig. 4:**
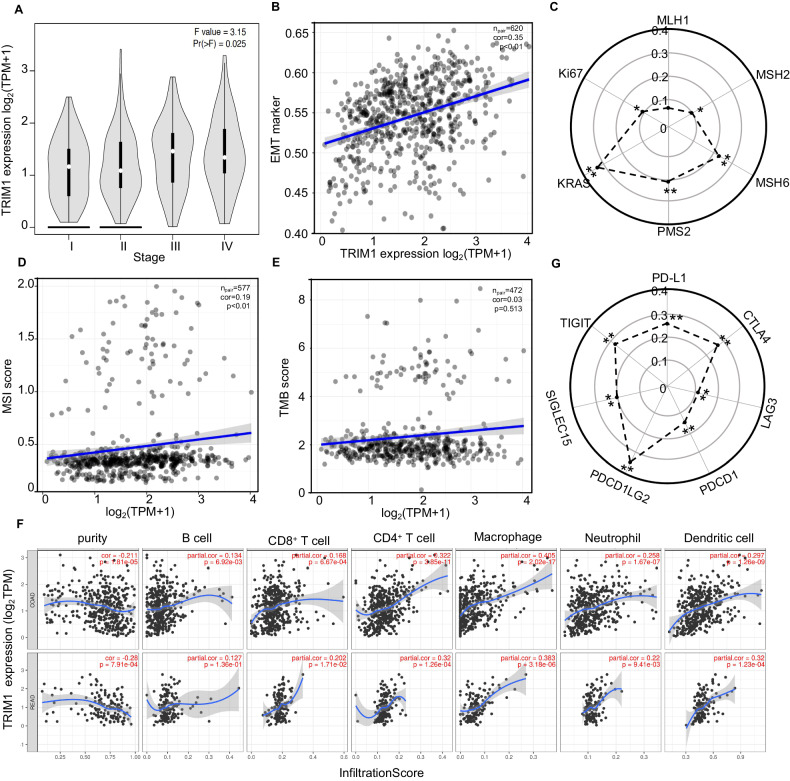
Higher expression of TRIM1 was significantly associated with poor prognostic and immunotherapy biomarkers in colorectal cancer. **A** TRIM1 expression in different CRC pathological stages using TCGA data. **B** Correlation between TRIM1 expression and EMT marker by GESA enrichment analysis of CRC patients’ data. **C** Correlation between TRIM1 expression and MMR genes in CRC patients. **D**, **E** Correlation analysis between TRIM1 expression and MSI/TMB score of CRC patients. The abscissa represents gene expression distribution, and the ordinate represents MSI (**D**) and TMB (**E**) score distribution. The value in the panel represents the paired-sample number, correlation coefficient, and correlation *p* value. **F** Correlation between TRIM1 expression and immune cell infiltration levels in COAD and READ. **G** Correlation between the TRIM1 expression and immune checkpoint genes. Spearman’s correlation coefficients and *p* values were shown. *p < 0.05, **p < 0.01.

To explore the potential roles of TRIM1 in CRC development, we next analyzed the relationship between TRIM1 mRNA expression level and its clinical outcomes. We observed positive correlations between the expression level of TRIM1and the CRC tumor stage (Fig. 4A), the EMT signaling (Fig. 4B), and two malignant tumor marker genes Ki67 and KRAS (Fig. 4C), implying that TRIM1 may play a promotive role in CRC tumorigenesis.

Growing studies have proved that microsatellite instability high MSI status (MSI-H) of mismatch repair deficient (dMMR) gene may predict immunotherapeutic response in CRC. dMMR-MSI-H signatures are typically closely related to the high tumor mutation burden (TMB-H) or immune cell infiltration [11, 12]. To determine the potential role of TRIM in immunotherapeutic response, we performed correlation analyses using the TCGA RNA-seq data of CRC samples. The TRIM1 mRNA level highly correlated with the dMMR-MSI-H signature in CRC samples, including three MMR genes (MSH2, MSH6, and PMS2) and MSI score (Fig. 4C, D). TRIM1 expression had non-significant correlations with TMB but showed positive correlation with infiltrating levels of immune cells (CD8^+^ T cells, CD4^+^ T cells, macrophage, neutrophils, and dendritic cells) in CRC (Fig. 4E, F). Consistently, TRIM1 mRNA level had dramatically positive coefficients with the canonical immune checkpoint genes (Fig. 4G). Together, these results elucidated the possible role of TRIM1 in regulating immunotherapeutic response in CRC.

### TRIM1 promotes cell proliferation of CRC

**Fig. 5:**
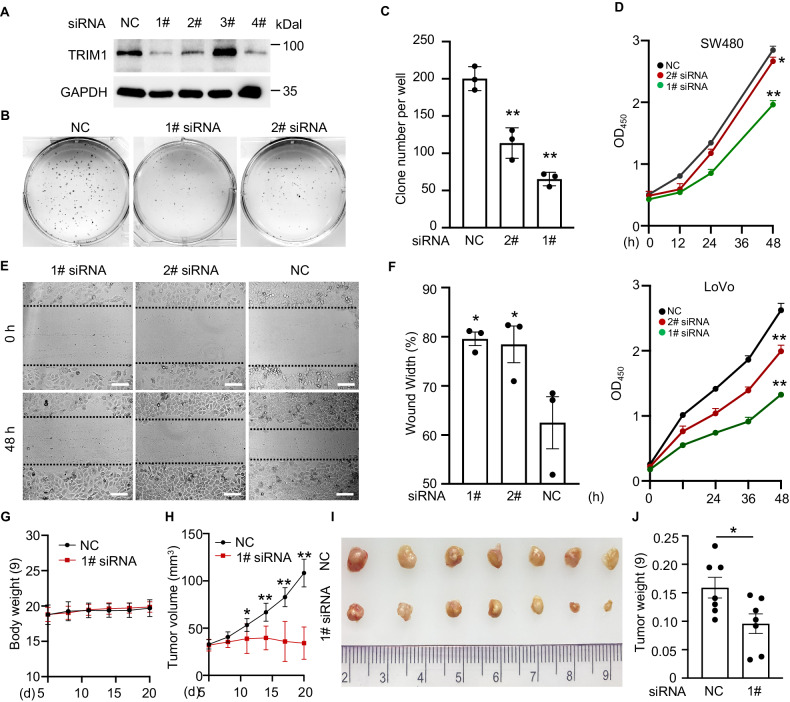
TRIM1 silencing attenuates the colony formation, proliferation, and migration of colorectal cancer cells. **A**–**F** Colorectal cancer cells were transfected with TRIM1 siRNA for 48 h, and then subjected to the colony formation assay, cell proliferation assay, or wound scratch assay. **A** Knockdown efficiency of TRIM1 siRNA was detected by immunoblotting. **B**, **C** Effects of TRIM1 knockdown on the colony formation of SW480 cells. Representative images were shown (**B**), and the colony number was calculated (**C**). **D** Effects of TRIM1 knockdown on the cell proliferation of SW480 and LoVo cells. **E**, **F** Effects of TRIM1 knockdown on the migration of SW480 cells. Representative images were shown (**E**), and the wound width was calculated (**F**). **G**–**J** Effects of TRIM1 knockdown on the growth and proliferation of SW480 cells in the nude mice xenograft model. Effects of TRIM1 knockdown on the tumor growth. Measurement was conducted every 3 days (**G**, **H**). The tumor volume and weight were measured on the 20th day after tumor inoculation (**I**, **J**). Results are as means ± SD from three independent experiments. Scale bar, 100 μm. *p < 0.05, **p < 0.01.

Clinical analyses implied that TRIM1 played a tumor-promoting role in CRC, so we next examined the biological functions of TRIM1 in CRC cells. We synthesized four pairs of siRNAs for the loss-of-function study and found that the first and the second pairs showed an excellent silencing effect (Fig. 5A). Also, for the gain-of-function study, we constructed the functional pCS2-GFP-plasmid expressing the wild-type (WT) TRIM1 and the catalytically inactive mutant ΔRING TRIM1 for over-expression in CRC cells. Overexpression of TRIM1 in SW480 and LoVo cells dramatically increased the migration rate and the colony formation of CRC cells compared with the corresponding controls (Supplementary Fig. S1). Silencing of TRIM1 efficiently decreased the colony number of SW480 cells (Fig. 5B, C), attenuated the cell proliferation both in SW480 and LoVo cells (Fig. 5D), and slowed down the migration rate (Fig. 5E, F). Notably, this inhibition effect was not due to cell death because TRIM1 siRNA treatment did not induce apparent cell death based on the detection of the lactate dehydrogenase (LDH) release and the caspase-3 activity with or without the treatment of the apoptosis stimuli cisplatin (Supplementary Fig. S2). Next, we examined whether TRIM1 affects tumorigenesis in vivo. No significant body weight was lost during the TRIM1 siRNA, suggesting that TRIM1 was not overtly toxic in vivo (Fig. 5G). We observed that TRIM1 silencing can efficiently inhibit the growth and proliferation of SW480 cells in the nude mice xenograft model (Fig. 5H). Under treatment with TRIM1 siRNA, the tumor volumes and weights were significantly lower (Fig. 5I, J). Collectively, the above data demonstrated an oncogenic role of TRIM1 in CRC.

### TRIM1 facilitates metabolism and restrains immune response

**Fig. 6:**
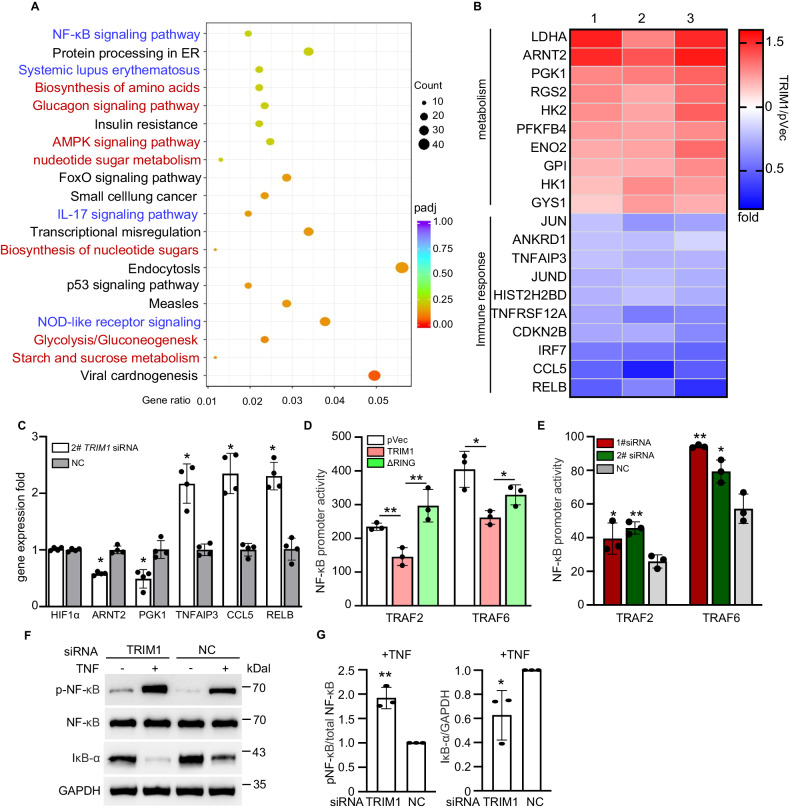
TRIM1 is critical for metabolism promotion and immune suppression. **A**, **B** Systematic RNA-seq analysis from TRIM1-overexpressed SW480 cells. **A** Pathway enrichment of the DEGs by GO and KEGG analyses using the DAVID online tool. The top 20 pathways were listed. The circle size represents the number of DEGs enriched in this pathway. Red labels correspond to the upregulated pathways, and blue labels refer to the downregulated pathways. **B** Heatmap shows the synergistic expression patterns of the DEGs involved in regulating metabolism and immune response post-TRIM1 transfection. Color change from blue to red represents the expression levels of DEGs from low to high. **C** Effects of TRIM1 silencing on the expression of genes involved in regulating metabolism and immune response. After siRNA treatment for 48 h, the expression of the selective genes was examined by qRT-PCR. Primers were displayed in Supplementary Table S2. **D**, **E** Effects of TRIM1 overexpression and silencing on the NF-κB activities. **D** The plasmid for GFP-WT TRIM1, GFP- ΔRING TRIM1 or GFP was co-transfected with the plasmid constructs for NF-κB-Luc, adapter molecule TRAF2 or TRAF6. **E** After transfection of TRIM1 siRNA for 48 h, SW480 cells were co-transfected with plasmid constructs for NF-κB-Luc, adapter molecule TRAF2 or TRAF6 into SW480 cells. NF-κB activity in these samples was determined using the luciferase reporter assay. **F**, **G** Effects of TRIM1 knockdown on the NF-κB pathway. After siRNA treatment for 48 h, SW480 cells were added with TNF for another 12 h. **F** The expression of NF-κB pathway-related protein was examined by immunoblotting. **G** The level of NF-κB pathway activation was quantitated by measuring the ratio of band signal intensity for phosphorylated NF-κB/ total NF-κB, and IκB/GAPDH with Image J. Results are as means ± SD from three independent experiments. *p < 0.05, **p < 0.01.

Our results indicate TRIM1 as an essential factor in promoting the proliferation of CRC cells. To investigate the crucial roles of TRIM1 in genome-wide gene expression changes and intracellular signaling pathways, we conducted a systematically transcriptional analysis of TRIM1-transfected SW480 cells was performed. Based on the RNA-seq analyses, TRIM1 transfection in SW480 cells led to the upregulation of 736 genes and the downregulation of 961 genes (Supplementary Fig. S3A). These DEGs were assigned to GO/KEGG analyses, and the top 20 enriched pathway lists were shown. The functions were primarily divided into positive regulation of metabolism (in red) and negative regulation of innate immune (in blue) (Fig. 6A and Supplementary Fig. S3B, C). The heat map showed the upregulation of critical metabolic genes and the downregulation of immune-related genes (Fig. 6B). Consistently, TRIM1 silencing by siRNA oligonucleotides results in the increased mRNA level of immune-related genes (TNFAIP3, CCL5, and RELB) and the decreased mRNA level of metabolic genes (ARNT2 and PGK1) (Fig. 6C).

To verify the roles of TRIM1 in the negative regulation of inflammation in vitro, we examined the canonical NF-κB pathway by NF-κB-luciferase assay and immunoblotting. Over-expression of FL TRIM1, but not ΔRING TRIM1, in SW480 significantly decreased the TRAF2/TRAF6-mediated NF-κB activity (Fig. 6D). Conversely, TRIM1 silencing by siRNA oligonucleotides results in an elevated NF-κB activity (Fig. 6E). Besides, TRIM1 knockdown increased the endogenous level of NF-κB phosphorylation and IκBα degradation induced by TNF, confirming the TRIM1-mediated NF-κB pathway blockade (Fig. 6F, G).

### TRIM1 interacts with and catalyzes K63-linked ubiquitination on HIF1α

**Fig. 7:**
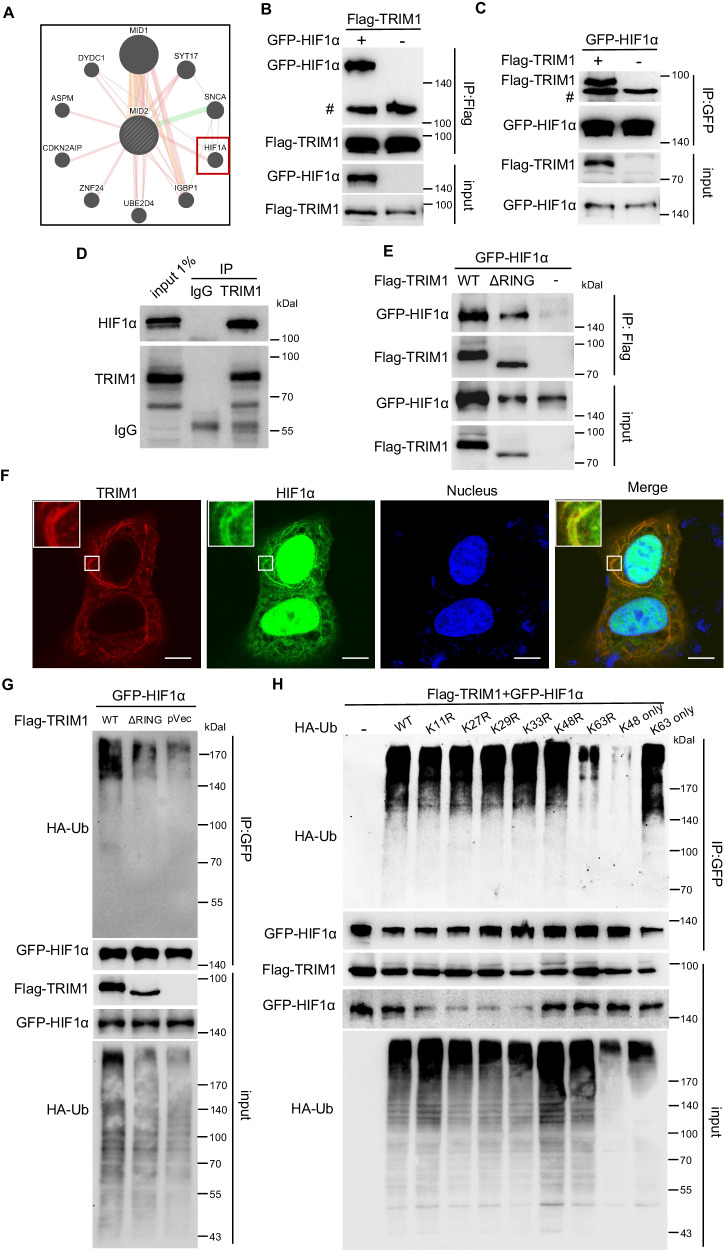
TRIM1 interacts with HIF1α and catalyzes its K63-linked ubiquitination. **A** The protein–protein interaction network (PPI) of MID2/TRIM1 by GeneMANIA. Shown are the top 10 most related proteins. **B**–**E** The interaction between TRIM1 and HIF1α by coimmunoprecipitation (co-IP) assay. SW480 cells were co-transfected with the indicated plasmids. Samples lysed were immunoprecipitated with anti-Flag, anti-GFP or anti-TRIM1 antibody, and the input and immunoprecipitated samples were detected by immunoblotting with the indicated antibodies. **B**, **C** Overexpressed TRIM1 and HIF1α were co-immunoprecipitated with each other. **D** Endogenous TRIM1 and HIF1α can be co-immunoprecipitated. **E** ΔRING TRIM1 was coimmunoprecipitated with HIF1α. **F** HIF1α co-localized with TRIM1 at microtubules. SW480 cells were co-transfected with GFP-HIF1α and Flag-TRIM1 plasmids for 18 h. Shown are photos of the cellular localization of HIF1α (green) and TRIM1 (red). Scale bar, 10 μm. **G** Overexpression of TRIM1 promotes ubiquitination of HIF1α. GFP-HIF1α expressed-SW480 cells were transfected with the empty control vector or a plasmid expressing WT TRIM1 or ΔRING TRIM1 in the presence of the WT HA-ubiquitin. At 18 h post-transfection, GFP-HIF1α was immunoprecipitated with an anti-GFP antibody, followed by immunoblotting analysis with the corresponding antibodies. **H** TRIM1 catalyzes K63-linked polyubiquitination of HIF1α. GFP-HIF1α expressed-SW480 cells were transfected with Flag-TRIM1 plasmid or the empty control vector in the presence of the WT and mutated HA-ubiquitin. 18 h post-transfection, GFP-HIF1α was immunoprecipitated with an anti-GFP antibody, followed by immunoblotting analysis with the corresponding antibodies.

To further understand the molecular mechanism underlying the signaling pathways related to TRIM1 in CRC, we next analyzed the direct Protein interaction network (PPI) to determine potential interaction baits of TRIM1 (also called MID2). Besides the well-studied microtubule-binding protein MID1 and the ubiquitin-conjugating enzyme E2 D4 UBE2D4, we were surprised to find that TRIM1 was closely associated with the transcription factor hypoxia-inducible factor-1α (HIF1α) (Fig. 7A). Coimmunoprecipitation (co-IP) assay showed that over-expressed and endogenous TRIM1 and HIF1α could interact, which was detected by immunoblotting (Fig. 7B–D). Deletion of the N-terminal RING domain did not abolish this interaction (Fig. 7E). Also, TRIM1 was observed to co-localize with HIF1α at microtubules by confocal microscopy (Fig. 7F). TRIM1 is an E3 Ub ligase, and we next evaluated whether TRIM1 ubiquitinated HIF1α in vivo. Compared with the control plasmid, co‐transfection of FL TRIM1 with HIF1α results in robust ubiquitination of HIF1α, while expression of the enzymatically inactive TRIM1 did not induce additional ubiquitination (Fig. 7G). Besides, we used a series of lysine mutants of Ub to determine the poly-Ub chain type on HIF1α. Strong ubiquitination of HIF1α appeared in reactions containing wild-type (WT), K11R, K27R, K29R, K33R, or K63-only Ub (a mutant in which all Lys residues have been mutated to Arg residues except for Lys63). However, in the sample with the K63R or K48-only ubiquitin mutant, ubiquitination was largely inhibited (Fig. 7H). Thus, our data suggested that TRIM1 interacted with HIF1α on microtubules and accelerated its K63-conjugated ubiquitination.

### Lys214 of HIF1α is ubiquitinated by TRIM1 and is essential for HIF1α’s activity

**Fig. 8:**
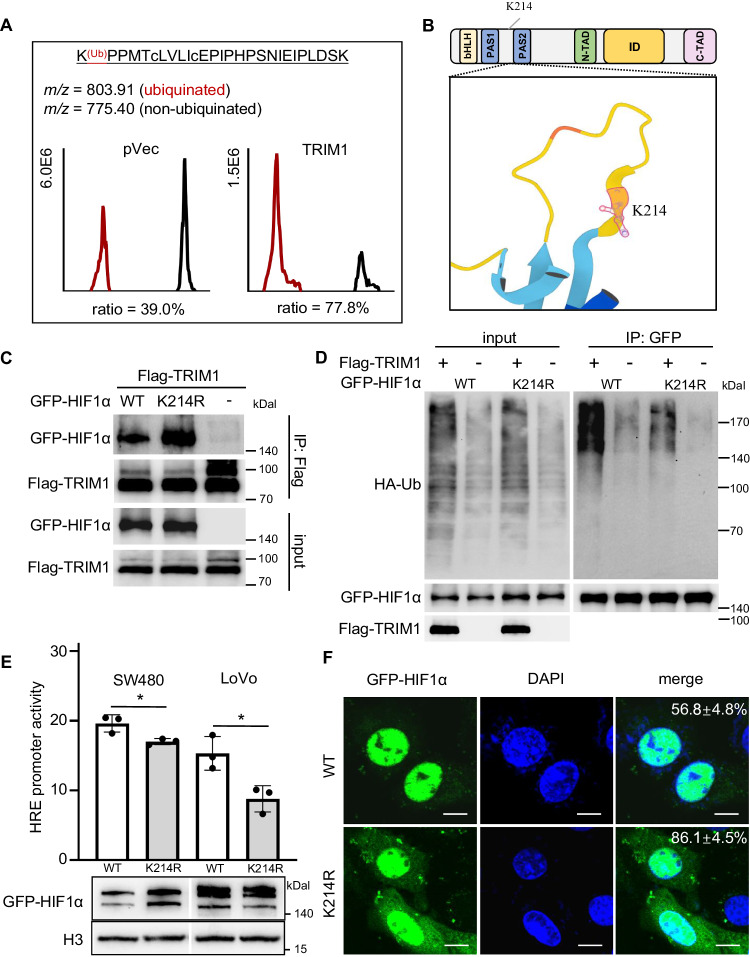
Lysine 214 of HIF1α is ubiquitinated by TRIM1 and is essential for HIF1α’s activity. **A** Mass spectrometric detection of modified peptides of HIF1α. Extracted ion chromatograms are shown with peak intensities indicating the relative amounts of either the modified or unmodified peptides of HIF1α in the presence/absence of TRIM1. The intensity of peptides of HIF1α is listed in Supplementary Table S1. **B** The protein motif and 3D structure visualization of K214 in HIF1α. The complete structure of HIF1α is currently unavailable and predicted by the AlphaFold online tool (https://alphafold.ebi.ac.uk/). **C** The interaction between TRIM1 and the WT or K214R HIF1α by Co-IP assay. SW480 cells were co-transfected with the indicated plasmids. Samples lysed were immunoprecipitated with anti-Flag antibody, and the input and immunoprecipitated samples were detected by immunoblotting with the indicated antibodies. **D** Validation of K214 as a ubiquitination modification site of HIF1α by TRIM1. Flag-TRIM1 expressed-SW480 cells were transfected with the empty control vector or a plasmid expression WT HIF1α or K214R HIF1α in the presence of the WT HA-ubiquitin. At 18 h post-transfection, GFP-HIF1α was immunoprecipitated followed by immunoblotting analysis with the corresponding antibodies. **E** Effect of K214 mutation on the HIF1α activity. The plasmid construct for WT or K214R GFP HIF1α was co-transfected with the plasmid constructs for HRE-Luc into SW480 cells. HIF1α activity was determined using a luciferase reporter assay. The expression was shown below. *p < 0.05. **F** Effect of K214 mutation on the nucleus distribution of the HIF1α. The plasmid construct for WT or K214R GFP HIF1α was transfected into SW480 cells under normoxic conditions for 18 h. Shown are representative images showing cellular localization of HIF1α (green) and nucleus (blue). The percentage of GFP-HIF1α in the cytosol for each sample is indicated. At least 50 cells were counted for samples from experiments done in triplicate. Scale bar, 10 μm.

To precisely map the modification site(s), we affinity-purified GFP-HIF1α from SW480 cells co-transfected with either wild-type TRIM1 or empty vector. From quantitative mass spectrometry, we detected six ubiquitination peptides of HIF1α. Our data reveal that peptide -^214^KPPMTcLVLIcEPIPHPSNIEIPLDSK^240^- was highly (~77.8%) ubiquitinated in the presence of TRIM1 (Fig. 8A). In contrast, the change of modification rate for the other modified peptides was below 10% (Supplementary Table S1). MS/MS analyses assigned the major modification site to Lys214 (K214) (Fig. 8A). K214 is predicated to be located within the loop between the two helix Per-ARNT-Sim (PAS) domains of HIF1α (Fig. 8B). Mutation of Lys214 to Arg of HIF1α did not abolish the binding with TRIM1 (Fig. 8C). However, the ubiquitination signals were significantly attenuated in samples expressing the HIF1α K214R mutant (Fig. 8D). Upon activation, the transcription factor HIF1α is translocated to the nucleus and binds the consensus HREs (hypoxia-responsive element) in the target gene promoter regions to initiate expression [13]. To mimic the HIF1α activity in vitro, we applied an HRE-luciferase reporter. Compared with the WT HIF1α, K214R decreased activity and displayed less nuclear localization under normoxic condition in CRC cells (Fig. 8E, F).

### TRIM1 promotes HIF1α activity by accelerating its nuclear translocation

**Fig. 9:**
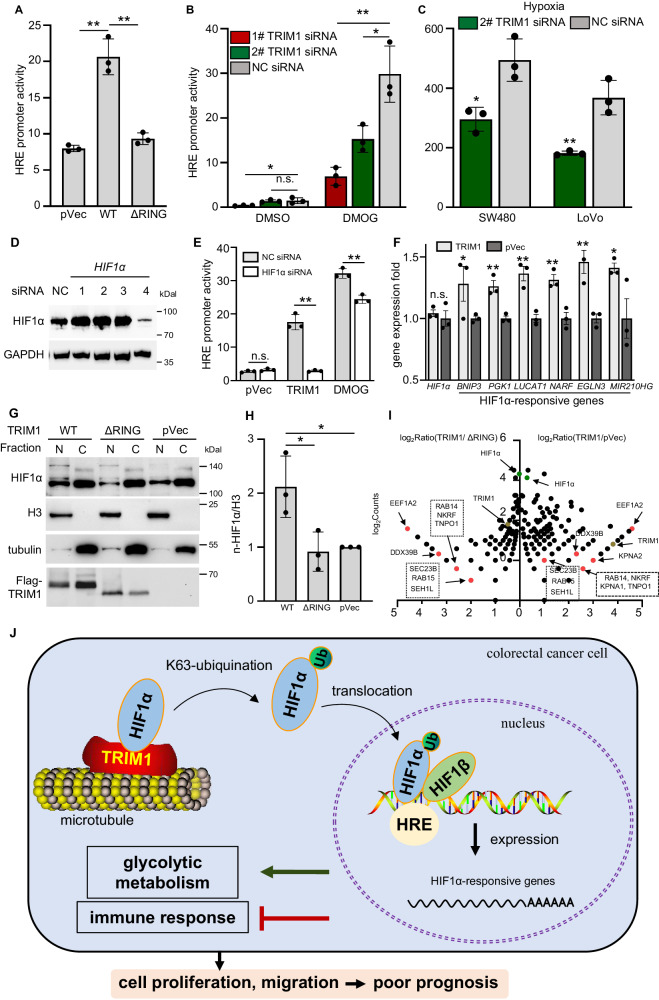
TRIM1 promotes HIF1α activity by accelerating its nuclear translocation. **A** Effects of TRIM1 over-expression on the HIF1α activities. The plasmid construct for WT GFP-TRIM1, ΔRING GFP-TRIM1, or GFP was co-transfected with the plasmid constructs for HRE-Luc into SW480 cells. HIF1α activity was determined using a luciferase reporter assay. **B** Effects of TRIM1 knockdown on the DMOG-induced HIF1α activities. After transfection of TRIM1 siRNA for 48 h, SW480 cells were transfected with the HRE-Luc plasmid in the presence or absence of DMOG. **C** Effects of TRIM1 knockdown on the hypoxia induced HIF1α activities. After transfection of TRIM1 siRNA for 48 h, SW480 and LoVo cells were transfected with the HRE-Luc plasmid. After 10 h, cells were subjected to the hypoxic treatment (1% O_2_, 5% CO_2_, 95% humidity) for another 18 h. HIF1α activity was determined using a luciferase reporter assay. **D**, **E** Effects of HIF1α knockdown on the TRIM1-mediated HRE promoter activity. **D** The silencing efficiency of HIF1α siRNA was determined by immunoblotting. **E** After transfection of 4# HIF1α siRNA for 48 h, HRE-Luc plasmid was co-transfected with a plasmid construct for GFP-TRIM1 or GFP into SW480 cells. DMOG treatment acted as the positive control. **F** Effects of TRIM1 expression on the mRNA expression of HIF1α and the HIF1α*-*responsive genes. The figure was generated from our transcriptome data. **G**, **H** Effects of TRIM1 over*-*expression on the nucleus distribution of the endogenous HIF1α. **G** SW480 cells were transfected with a plasmid for WT Flag- TRIM1, ΔRING Flag-TRIM1 or GFP for 18 h. Total nucleus (N) and cytosol proteins (C) were fractionated and immunoblotted with the indicated antibodies. **H** The nuclear distribution of HIF1α was quantitated by determining the ratio of band signal intensity for HIF1α/ H3 in (**G**) with Image J software. **I** Quantification of the TRIM1-mediated HBPs in SW480 cells. GFP-HIF1α were co-expressed with Flag-tagged WT TRIM1, ΔRING TRIM1 or pVec into SW480 cells for 18 h. Lysates were subjected to IP with GFP-specific antibody. The precipitates were further separated by SDS-PAGE before in-gel digestion with trypsin and LC-MS/MS analyses. Scatter plots of protein ratios as a function of their relative abundance (denoted by MS/MS spectral counts). The ratio is calculated as spectral counts in FL TRIM1 transfected samples divided by those in controls. Higher ratios indicate increased binding efficiency with HIF1α. Red dots correspond to the potential HBPs involved in nuclear import, the green dots correspond to immunoprecipitated HIF1α, and the yellow dots correspond to TRIM1. Results are as means ± SD from three independent experiments. *p < 0.05, **p < 0.01. **J** Schematic diagram of this work. TRIM1 expression promotes the proliferation and migration of colorectal cancer cells and predicts poor prognosis for CRC patients. Mechanistically, TRIM1 interacts with HIF1α, catalyzes its K63-linked ubiquitination, and promotes its nuclear translocation. HIF1α in the nuclear then binds the HRE region in the promoter, initiates the expression of downstream genes, promotes cellular metabolism, and attenuates immune response.

Then, we sought to determine the consequences of HIF1α ubiquitination by TRIM1. Over-expression of the WT but not the enzymatically active TRIM1 significantly elevated the HRE activity (Fig. 9A). Conversely, TRIM1 knockdown by siRNA oligonucleotides results in an attenuated HRE activity induced by DMOG (a HIF1α activator) and hypoxic treatment (Fig. 9B, C). Knockdown of HIF1α significantly decreased HRE activity induced by TRIM1 and DMOG (Fig. 9D, E). Besides, our transcriptome results showed the increased expression of HIF1α-downstream genes in the TRIM1-transfection sample (Fig. 9F), confirming TRIM1-mediated HIF1α activation. Although several E3 ligases have been reported to regulate HIF1α’s activity via alteration of its expression level or protein stability, our results showed that TRIM1 expression or silencing did not alter the HIF1α mRNA level (Figs. 9F and 6C). Chase experiments with cycloheximide (CHX) showed that TRIM1 expression also did not affect the protein stability of HIF1α (Supplementary Fig. S4). Interestingly, WT TRIM1 overexpression led to the nucleus translocation of endogenous HIF1α (Fig. 9G, H) after nucleus and cytoplasmic fractionation. These results suggest that TRIM1 activates HIF1α signaling by accelerating its nucleus translocation instead of altering its expression.

### TRIM1 facilitates the association of HIF1α with nucleus transport proteins

To investigate the molecular mechanisms underlying TRIM1-mediated HIF1α nucleus translocation, we used mass spectrometry to analyze proteins pulled down by HIF1α with and without TRIM1 (Supplementary Fig. S5A). HIF1α interactome analyses showed that the HIF1α-binding proteins (HBPs) were more abundant in TRIM1-transfected samples. Among these, 308 differential HBPs overlapped in TRIM1/ΔRING and TRIM1/pVec groups (Supplementary Fig. S5B). KEGG analysis of the putative HBPs revealed several enriched metabolic pathways (Supplementary Fig. S5C). Interestingly, seven proteins are involved in the nucleocytoplasmic transport pathway (in red), including DDX39B, SEH1L, EEF1A2, NUP153, NUP214, NUP98, and TNPO1 (Fig. 9I). Consistent with this notion, we also determined the effects of the modification site (K214) of HIF1α on the HBPs and obtained similar HIF1α interactome results. When K214 was mutated, there was also a drastic reduction in the redundancy of these HBPs (Supplementary Fig. 5D, E). Therefore, we speculate that TRIM1 potentially enhances the interaction of HIF1α with nuclear transport proteins through ubiquitination at K214 of HIF1α, increasing its probability of entering the nucleus.

## Discussion

Increasing evidence has demonstrated that TRIM proteins play crucial roles in regulating tumorigenesis [14]. TRIM1 is a special E3 ligase at the microtubule involved in cytokinesis and cell division [15, 16]. Two noteworthy reports have shown that a high level of TRIM1 is related to increased chemoresistance and poor prognosis in breast cancer cells [17, 18]. However, TRIM1’s associations with the clinical significance, biological functions, and molecular mechanism in carcinogenesis remain unknown. In this study, we demonstrated that TRIM1 expression is positively associated with CRC pathological stages, and higher expression is positively correlated with immunotherapy biomarkers and poor prognosis. TRIM1 markedly promotes CRC cell migration, proliferation, and colony formation in cultured cells. Combined with a systematically transcriptional analysis, we revealed the involvement of TRIM1 in boosting metabolism and inhibiting immune response. Mechanistically, TRIM1 could bind HIF1α to promote its ubiquitination and mediate its nuclear translocation and activation (Fig. 9J). Together, our findings provided TRIM1’s associations with clinical significance and demonstrated the novel oncogenic role of TRIM1 in CRC via activation of HIF1α signaling.

Consistent with previous studies in cancer [17–19], our data also indicates that TRIM1 is an oncogene and predicts poor prognosis. However, there are conflicting results regarding its expression level in cancer. Wang et al. demonstrated that TRIM1 protein is overexpressed in breast cancer specimens through immunohistochemical analysis of six patients [18]. By exploring cancer datasets from TCGA and GEO databases, we and another group found that TRIM1 is downregulated in many cancer tissues compared to control (Fig. 3E). This conclusion was also verified by qPCR and IHC assay using 10 paired CRC specimens in this study. The difference may be partly due to the limited numbers of matched cancer and normal samples in each study. Interestingly, Roy et al. reported that the expression of TRIM1 is likely to be regulated by immune signals. After TNF treatment, the mRNA expression and the protein stability of TRIM1 were up-regulated [20]. The expression of TRIM1 appears unaffected by methylation level within its CpG sites [19]. Instead, STAT3, activated by many immune cytokine signaling pathways, could increase TRIM1 expression by directly binding to the potential STAT3-binding sites in the TRIM1 promoter [17]. Our analysis showed that TRIM1 expression is positively correlated with infiltrating levels of immune cells and immune checkpoint genes. Elevated expression of TRIM1 negatively regulates the canonical NF-κB pathway and increases the resistance to chemotherapy drugs [17]. Thus, we speculate that TRIM1 expression may be only induced when the cancer cell receives the immune signals from the tumor immune microenvironment (TIME), thereby contributing to tumor immune escape and sustained tumorigenesis. So, it is intriguing to further investigate the molecular mechanisms governing TRIM1 gene expression in cancer development.

HIF1α, a key transcription factor in cellular responses to hypoxia, has been implicated in cancer [21]. HIF1α is often overexpressed in cancer tissues and associated with patients’ poor clinical prognosis [22–25]. HIF1α promotes tumor progression by regulating the transcription of various genes involved in metabolic reprogramming, metastasis, chemotherapy resistance, and immunosuppression [21]. HIF1α signaling and its protein stability are tightly controlled by ubiquitination. The von Hippel Lindau (VHL) protein acts as an E3 ubiquitin ligase that targets HIF1α for proteasomal degradation under normoxic conditions [26]. Mutations in the *VHL* gene are common in colorectal carcinoma and result in the accumulation of HIF1α, leading to tumor angiogenesis [27]. Besides, E3 ligases have been implicated in modulating HIF1α stability in cancer independent of oxygen. For example, TRAF6 binds HIF-1α and mediates its K63-linked polyubiquitination, maintaining HIF1α’s stability and promoting colorectal cancer development [28]. Parkin interacts with HIF-1α and promotes HIF-1α degradation through ubiquitination, inhibiting breast cancer cell metastasis [29]. On the contrary, NEDD4L, in the presence of its chaperone protein14-3-3, targets HlF-1a for poly-ubiquitination and subsequent proteasome-mediated degradation, enhancing the anti-tumor effect of bevacizumab in colorectal cancer [30]. In this study, we identified TRIM1 as a novel E3 ligase for HIF-1α. Compared with other E3 ligases that can either promote HIF1α’s degradation or stabilization, TRIM1 activates HIF-1α signaling by promoting its nuclear translocations instead of influencing the expression level, expanding the role of HIF1α ubiquitination in cancer.

Microtubule stabilization promotes HIF1α’ nucleus translocation under hypoxia [31], but the exact molecular mechanisms remain unknown. A previous study reported that the microtubule-associated motor protein dynein interacts with HIF1α and facilitates HIF1α nucleus translocation. It is proposed that dynein-HIF1α recruits BICD and the nuclear pore complex (NPC) protein RANBP2 to mediate the cargo nucleus translocation [31, 32]. TRIM1 locates on microtubules and contributes to the microtubule stabilization. In this study, we observed TRIM1 co-localized with HIF1α on microtubules and mediated its K63-linked ubiquitination. K63-linked ubiquitination is reported to be able to act as a scaffold for the formation of large protein complexes [33, 34]. From our MS data, we observed that the activity of TRIM1 and the ubiquitination of HIF1α significantly facilitated the interactions of HIF1α with nucleocytoplasmic transport proteins (DDX39B, SEH1L, EEF1A2, NUP153, NUP214, NUP98, RANBP2 and TNPO1) and cargo trafficking proteins (RAB14/15, SEC23B and VPS4A) (Fig. 9I and Supplementary Fig. 5D). Thus, we speculate that the TRIM1-mediated ubiquitination enhances the formation of cargo complexes of HIF1α with its translocation regulation factors, thus promoting nucleus translocation.

Therefore, we propose a novel mechanism of colorectal tumorigenesis via HIF1α regulation by TRIM1, which could potentially give rise to a new strategy for treating colorectal cancer.

## Materials and methods

### Plasmids, antibodies, and reagents

For transient expression in mammalian cells, full open reading frames (ORF) for TRIM1 and HIF1α were amplified using a SW480 cDNA library and inserted into the pCS2-EGFP and pCS2-Flag vectors. pRK5-HA-Ub-WT and the lysine mutants plasmids were maintained in our lab [35]. HRE-luc, pNF-κB-Luc, and pRL-TK reporter plasmids were purchased from Addgene. The sequences of all plasmids were confirmed by sequencing before use.

Antibodies for GAPDH (G9545) and Flag (F7425) were purchased from Sigma-Aldrich. EGFP (sc8334) antibody was obtained from Santa Cruz Biotechnology. Antibodies for HIF1α antibodies (D1S7W, #36169S), NF-κB p65 (D14E12, #8242), phospho-NF-κB p65 (Ser536) (93H1, #3033) and IκBα (44D4, #4812) were from Cell Signaling Technology. Anti-TRIM1/MID2 (68359-1-Ig) and anti-HA Epitope Tag (901501) antibodies were from Proteintech and Biolegend. DMOG was from Selleckchem. Cell culture products were from Invitrogen. The relevant chemicals in this study were obtained from Sigma-Aldrich unless stated otherwise.

### Cell culture, transfection, and luciferase reporter assay

SW480 and LoVo cells were purchased from the American Type Culture Collection (ATCC). They were cultured in high-glucose Dulbecco’s modified Eagle Medium (DMEM, HyClone) supplemented with additional 10% fetal bovine serum (FBS, Gibco), 2 mM L-glutamine, 1% v/v penicillin/streptomycin. Cells were cultured in a humidified incubator with 5% CO_2_ at 37 °C. Transient transfection reaction was conducted with the Jetprime reagents (Polyplus) according to the manufacturers’ data sheets. For the siRNA silencing assay, 200 pmol of siRNAs were transfected into 2 × 10^6^ cells. Sense sequences for the effective siRNAs used in this study are displayed as follows: TRIM1 1# 5’-GCAGCTCTGGTGAATCCAT-3’, TRIM1 2#: 5’-GGTGAATACTGCT ATGCAT-3’, HIF1α 4# 5’-GGGATTAACTCAGTTTGAA- 3’, and negative control (NC): 5’-TTCTCCGAACGTGTCACGT-3’. Luciferase activity was measured using the dual luciferase assay kit (Promega) according to the manufacturer’s instructions.

### Colony formation assay

CRC cells were first transfected with plasmids for 18 h or siRNA for 48 h. Cells from each sample were re-digested with trypsin and were seeded in a 6-well cell culture dish (1000 cells per well). After a 2-week cultivation, cells were fixed with 4% paraformaldehyde (PFA) and subjected to crystal violet staining. The culture medium was refreshed every 5 days during incubation. Clone numbers were determined from three biological replicates.

### Xenograft tumor model

We constructed cell-derived subcutaneous xenograft (CDX) models to evaluate the effects of TRIM1 depletion in vivo. Four-week-old female BALB/c nude mice were purchased from the Experimental Animal Center, Hubei University of Medicine (Hubei, China). SW480 cells (1 × 10^7^) were injected subcutaneously into the flanks of each mouse. When the tumors became palpable (about 30 mm^3^ in size), the tumor-bearing mice were divided into two groups. Each group includes seven mice. All mice were selected and allocated randomly. No statistical methods were used to predetermine the sample sizes, no specific randomization method was used, and no blinding was performed in grouping. TRIM1 siRNA or control siRNA (1 nmol per injection, Genepharma) was intratumorally injected into the mice every 3 days. The siRNA for animals contains a combination of 2′F and 2′OMe modifications. The diameter and width of the tumors were measured every 3 days and used to calculate the tumor volumes using the formula *V* = 0.5 × *D* × *W*^2^ (*V*, volume; *D*, diameter; and *W*, width). All the mice were sacrificed at the appropriate time, and the tumors were removed, photographed, weighed, and embedded in paraffin for further analysis.

### Cell viability assay

Cell proliferation was determined by the Cell Counting Kit-8 (CCK-8) (#C0038, Beyotime). Briefly, CRC cells were treated with siRNA for 48 h, re-digested with trypsin, seeded into 96-well plates at a density of 1000 cells per well, and cultured for a certain time. Then, the cells were supplemented with 10 μL CCK-8 and maintained in the incubator for another 2 h. The data was obtained from a microplate reader by measuring the absorbance at 450 nm. For LDH release detection, the culture supernatant was collected for LDH measurement according to the manufacturer’s instructions (Cyto-tox96, Promega). All measurement results were derived from three independent biological triplicates.

### Wound scratch assay

Cell migration was determined using the in vitro scratch assay. Colorectal cells were cultivated on 6-well plates to approximate 80% confluence. The wound was introduced by scratching with a pipette tip on the monolayer cell. Cells were then gently washed twice with PBS and cultured in a serum-free culture medium. Wound images were captured at the indicated time to calculate the wound width and the closure rate.

### Caspase-3 activity assay

Caspase-3 activity was measured as described previously [36]. Briefly, equal volumes of cell lysates were mixed and incubated with reaction buffer (1 M sodium citrate, 10 mM dithiothreitol, and 50 mM Tris-HCl, pH 7.4) containing Ac-DEVD-AFC (20 μM final) for 30 min at 37 °C. Fluorescence signals were collected every 2 min for 1 h at *λ*_Exc_/*λ*_Em_ ≈ 405/510 nm.

### Cycloheximide (CHX) chase assays

At 18 h after transfection of the indicated plasmids, SW480 cells were treated with 100 μg/ml CHX before lysates were collected at different time points and analyzed by immunoblotting.

### SDS-PAGE and immunoblotting

Western-Blot (WB) assay was conducted according to our standard protocols. Briefly, cell lysates were mixed with 5×SDS loading buffer, boiled at 95 °C for 5 min, and then subjected to SDS-PAGE. Proteins were transferred to PVDF membranes and subjected to the following steps. Membranes were blocked for 30 min by 5% nonfat milk in TBST and then incubated with primary antibody for 1 h at room temperature. After three washes with TBST, membranes were incubated with the HRP-conjugated second antibody for 30 min. After another three washes, membranes were incubated in the chemiluminescent substrate, and the antibody-bound protein was detected using ChemiDoc (BioRad). Mouse primary antibodies were diluted according to the manufacturer’s instructions when used in immunoblotting.

### Immunoprecipitation and co-IP

SW480 cells were washed once with PBS and subsequently lysed in the pre-cooled buffer A (Buffer A: 25 mM Tris-HCl, pH 7.5, 150 mM NaCl, 10% glycerol, and 1% Triton X-100, supplemented with a protease inhibitor mixture). The lysates were pre-cleared and were subjected to anti-TRIM1, anti-Flag or anti-GFP immunoprecipitation according to the standard protocol. After four times washes with ice-cold wash buffer, the immunoprecipitates on the beads were eluted and denatured by boiling in the SDS-containing buffer at 95 °C for 5 min, followed by standard immunoblotting analysis.

### Immunofluorescence staining and confocal microscopy imaging

Immunofluorescence staining was performed following the standard protocols in our lab [37]. Briefly, cell samples were fixed with 4% PFA for 10 min, permeabilized with 0.2% Triton X-100 for 15 min, incubated with 2% bovine serum albumin (BSA) for a 30-min blockade, then incubated with the indicated primary antibody and subsequent Alexa Fluor-labeled secondary antibody (ThermoFisher). Fluorescence images were acquired under the confocal microscope (FV3000RS, Olympus). All image data shown are representative of randomly selected fields from at least five replicates.

### Immunohistochemistry (IHC) assay

The CRC and paired adjacent tissues were prepared into 3 mm paraffin sections. Each sample was subjected to a 10-minute deaffinity antigen retrieval by sodium citrate (pH 6.0), followed by incubation with mouse monoclonal TRIM1 antibody (1:100 dilution) or mouse control IgG and subsequent horseradish peroxidase (HRP) labeled secondary antibody. Afterwards, each section was subjected to staining with DAB reagent and counterstaining with hematoxylin. The immunoreactive score of the section was calculated as described previously [38].

### Transcriptomic analysis

SW480 cells were transfected with a plasmid expression GFP-TRIM1 or GFP. After 24 h, total RNA was isolated using TRIzol reagent (Invitrogen), and samples were subjected to RNA-seq at Novogene (Beijing, China). The reads were assigned to the genome sequences of *Homo sapiens*. Relative mRNA expression abundance was quantified by measuring the value of FPKM. The gene expression was considered reliable and significantly different only when the thresholds of *p* value reached −log_10_(*p* value) >1.3. Pathway enrichment of these differential expression genes (DEGs) was performed by GO and KEGG analyses using the DAVID online tool (https://david.ncifcrf.gov/).

### Bioinformatic analysis

The GEPIA online database (http://gepia.cancer-pku.cn/index.html) was used to analyze the mRNA expression of TRIMs between tumor and normal tissue and evaluate the associations between TRIM1 expression and prognosis value in CRC patients. TIMER (https://cistrome.shinyapps.io/timer/) was used to assess the associations between TRIM1 expression and immune cell infiltration levels in COAD and READ. GeneMANIA (http://www.genemania.org) was used to predict the potential binding proteins of TRIM1. Home for Researchers (https://www.home-for-researchers.com) was used to evaluate the correlation between TRIM1 expression and immune checkpoint genes, TMB, and MSI scores.

### MS analyses

To identify the ubiquitinated-containing peptides of HIF1α, purified GFP-HIF1α protein was subjected to digestion with trypsin, and the resulting peptides were separated on an EASY-nLC 1200 system (Thermo Fisher Scientific). The nano liquid chromatography gradient was as follows: 0–8% B in 3 min, 8–28% B in 42 min, 28–38% B in 5 min, and 38–100% B in 10 min (solvent A: 0.1% formic acid in water; solvent B: 80% CH3CN in 0.1% formic acid). Peptides eluted from the capillary column were applied directly onto a Q Exactive Plus mass spectrometer by electrospray (Thermo Fisher Scientific) for MS and MS/MS analyses. Mass spectrometry data were searched against the amino acid sequence of HIF1α and were performed with cleavage specificity allowing four miscleavage events. Mass spectrometry data were searched with the variable modifications of methionine oxidation, ubiquitin addition to lysine, and acetylation of protein N termini. For identification of the HIF1α-binding protein, immunoprecipitates were separated using SDS-PAGE, fixed, and visualized after silver staining as recommended by the manufacturer. An entire lane of bands was excised and subjected to in-gel trypsin digestion and MS/MS detections as described above. Identification of proteins was carried out using the Proteome Discoverer 2.2 program. Mass spectrometry data were searched against the Human proteomes depending on the samples with carbamidomethylation of cysteine set as a fixed modification. The precursor mass tolerance was set to 10 ppm, and the fragment mass tolerance was set to 0.02 Da. A maximum false discovery rate of 1.0% was set for protein and peptide identifications.

### Quantification and statistical analysis

Results are presented as mean ± SD (standard deviation) containing at least three biological replicates. The sample size was generally chosen based on preliminary data indicating the variance within each group and the differences between groups, and no data were excluded from the analysis. The variance is similar between the groups that are being statistically compared. Data were analyzed using a two-sided and unpaired Student’s *t* test to compare two experimental groups. A difference is considered significant as the following: *p < 0.05, **p < 0.01.

### Ethic approval and consent to participate

Study involving human material was performed in accordance with the Helsinki Declaration, and the protocol was approved by the Ethics Committee of the Affiliated Taihe Hospital of Hubei University of Medicine under the number 2023KS44. Written informed consent was obtained from each subject before the study. This research only involves the use of patient specimens and does not involve human research participants. A total of 4 paired CRC specimens (including tumor tissue and the matched normal tissue) and 6 paired paraffin-embedded tissue sections were provided by the Department of Pathology, Taihe Hospital.

All animal studies were approved by the Animal Ethics Committee of Hubei University of Medicine under the accession number 03124010W and have been carried out in accordance with the Declaration of Helsinki.

### Supplementary information

Supplementary Data

## Data Availability

The raw sequence data reported in this paper have been deposited in the Genome Sequence Archive in BIG Data Center, Beijing Institute of Genomics (BIG) (https://bigd.big.ac.cn/gsa) under the accession number HRA005825. The mass spectrometry proteomics data have been deposited to the ProteomeXchange Consortium (http://proteomecentral.proteomexchange.org) via the iProX partner repository with the data set identifier PXD046557. Other data supporting the findings of this study and Source data are included in the Supplementary Data and available upon reasonable request.
