# Supplementary material for: An E3 ligase TRIM1 promotes colorectal cancer progression via K63-linked ubiquitination and activation of HIF1α
Source: Oncogenesis. 2024 May 20;13(1):16. doi: 10.1038/s41389-024-00517-2 (PMC11106307; doi:10.1038/s41389-024-00517-2)
Supplement: Supplementary file 1 — Supplementary Data [file 41389_2024_517_MOESM1_ESM.pdf]

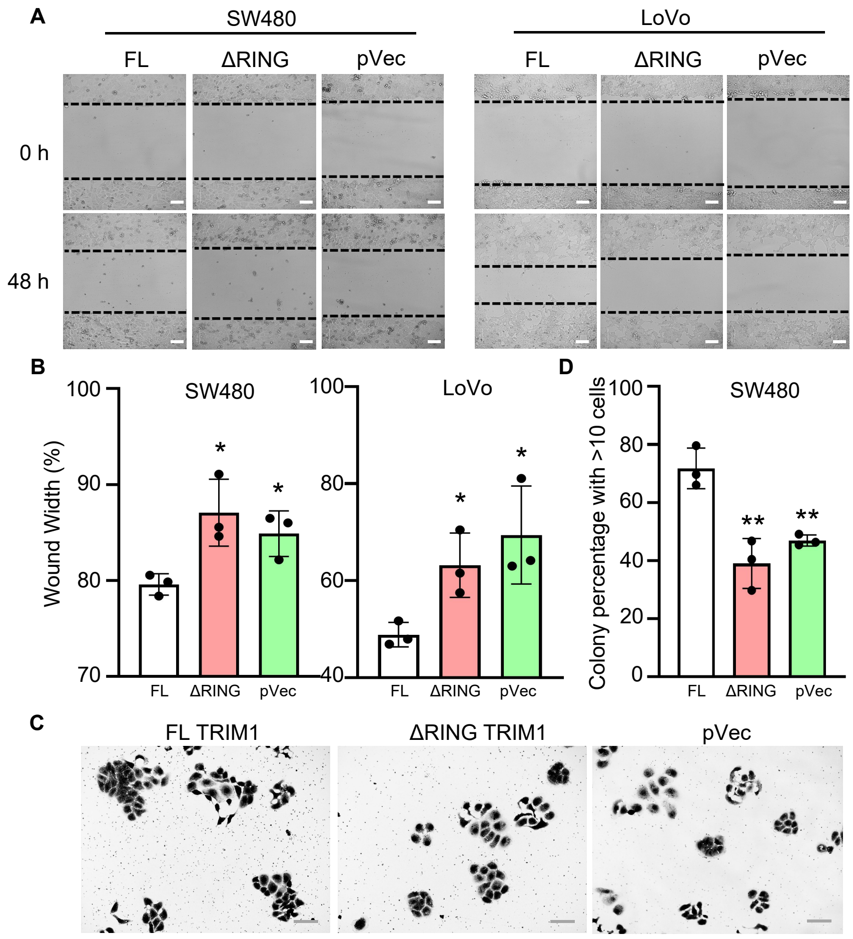

Supplementary Fig. S1: TRIM1 expression promotes the migration and colony formation of colorectal cancer cells. Colorectal cancer cells were transfected with a plasmid expressing GFP-TRIM1 FL(full lenth), ΔRING mutant or GFP (pVec) for 18 h, and then subjected to wound scratch assay and colony formation assay.

A-B Effects of TRIM1 over-expression on the migration of SW480 and LoVo cells. Representative images were shown (A), and the wound width was calculated (B).

C-D Effects of TRIM1 over-expression on the colony formation of SW480 cells. Representative images were shown (C), and the colony number was calculated (D).

Results are as means ± SD from at three independent experiments. *$p<0.05$, **$p<0.01$. Scale bar, 100 μm.

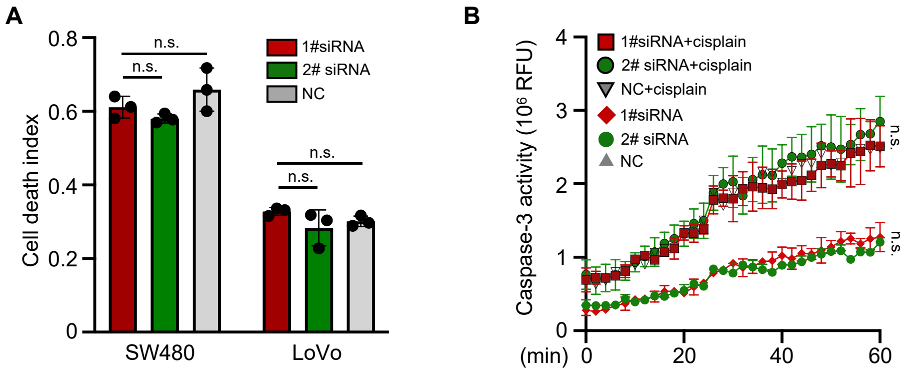

Supplementary Fig. S2: TRIM1 knockdown has no significant role for the cell death of CRC cancer cells.

A Effects of TRIM1 knockdown on the cell death of SW480 cells and LoVo cells. Cells were transfected with TRIM1 siRNA for 48 h, and then subjected to LDH assay.

B Effects of TRIM1 knockdown on the caspase-3 activity of SW480. Cells were transfected with TRIM1 siRNA for 48 h, treated with apoptosis stimuli cisplain for another 12 h, and then subjected caspase assay.

Results are as means ± SD from at three independent experiments. n.s., not significant.

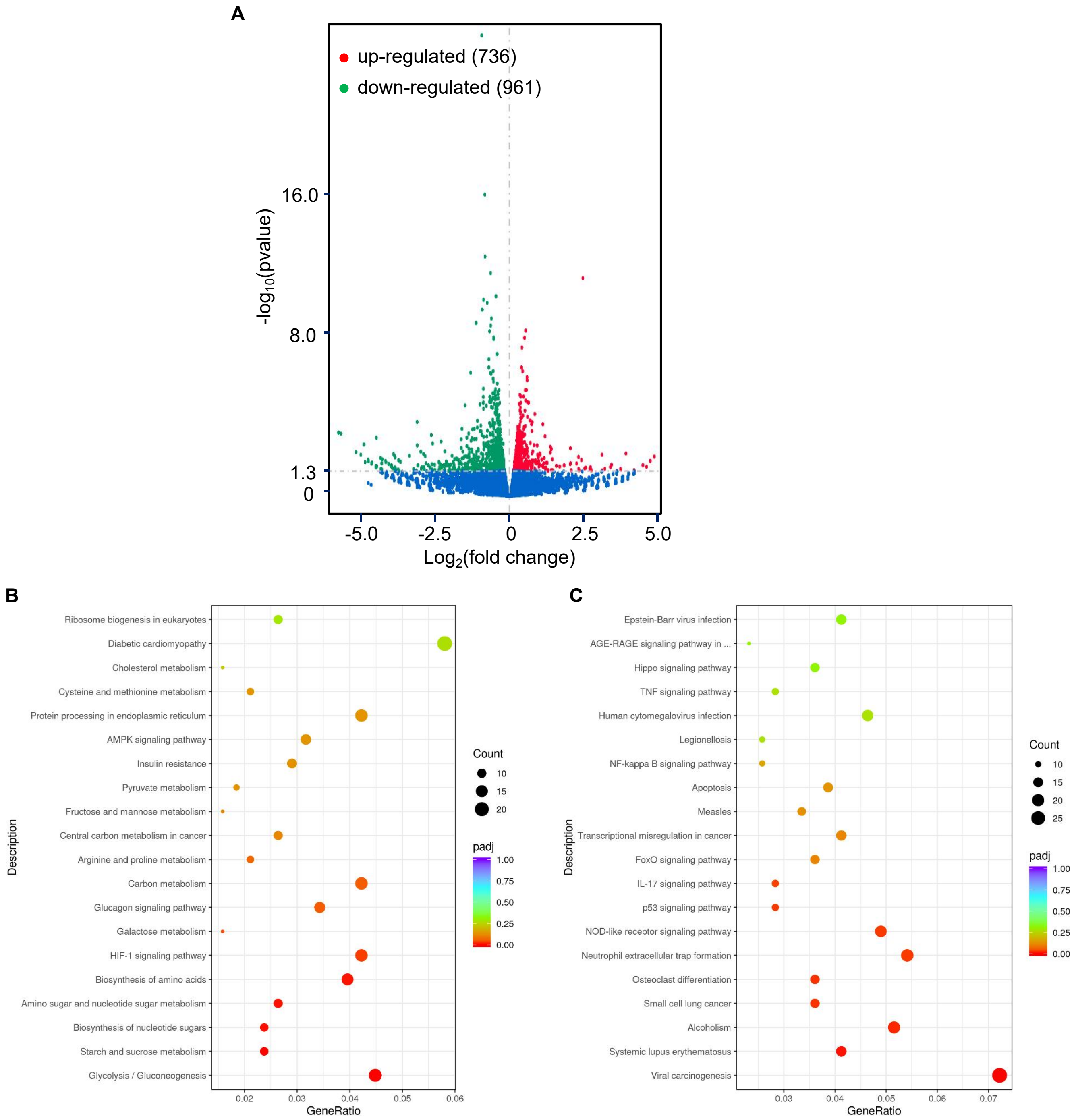

Supplementary Fig. S3: RNA-seq analysis from TRIM1-overexpressed SW480 cells.

A Volcano map showing the overall transcriptional expression in SW480 cells at 18 h post-transfection. Red dots represent the upregulated genes, green dots correspond to the downregulated genes, and blue dots represent insignificant genes.

B-C Gene Ontology enrichment analysis of the DEGs into biological processes using the DAVID online analysis tool. Top 20 up-regulated (B) and down-regulated (C) were listed. The big circle indicates a more significant number of DEGs enriched in this function. This figure responses to Figure 6.

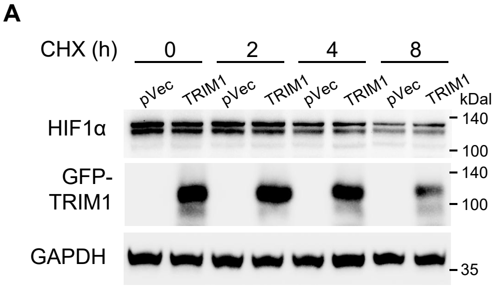

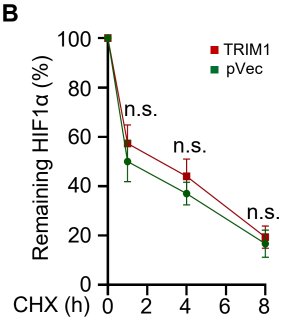

Supplementary Fig. S4: Effects of TRIM1 over-expression on the stability of the endogenous HIF1α.

A SW480 cells were transfected with a plasmid for GFP-TRIM1 or GFP for 18 h and subjected to CHX chase assay and standard immunoblotting analysis with the corresponding antibodies.

B The percentage of the remaining HIF1α was quantitated by determining the ratio of band signal intensity for HIF1α (indicated time)/ HIF1α (0 h) with Image J software.

Results are as means ± SD from at three independent experiments. n.s., not significant.

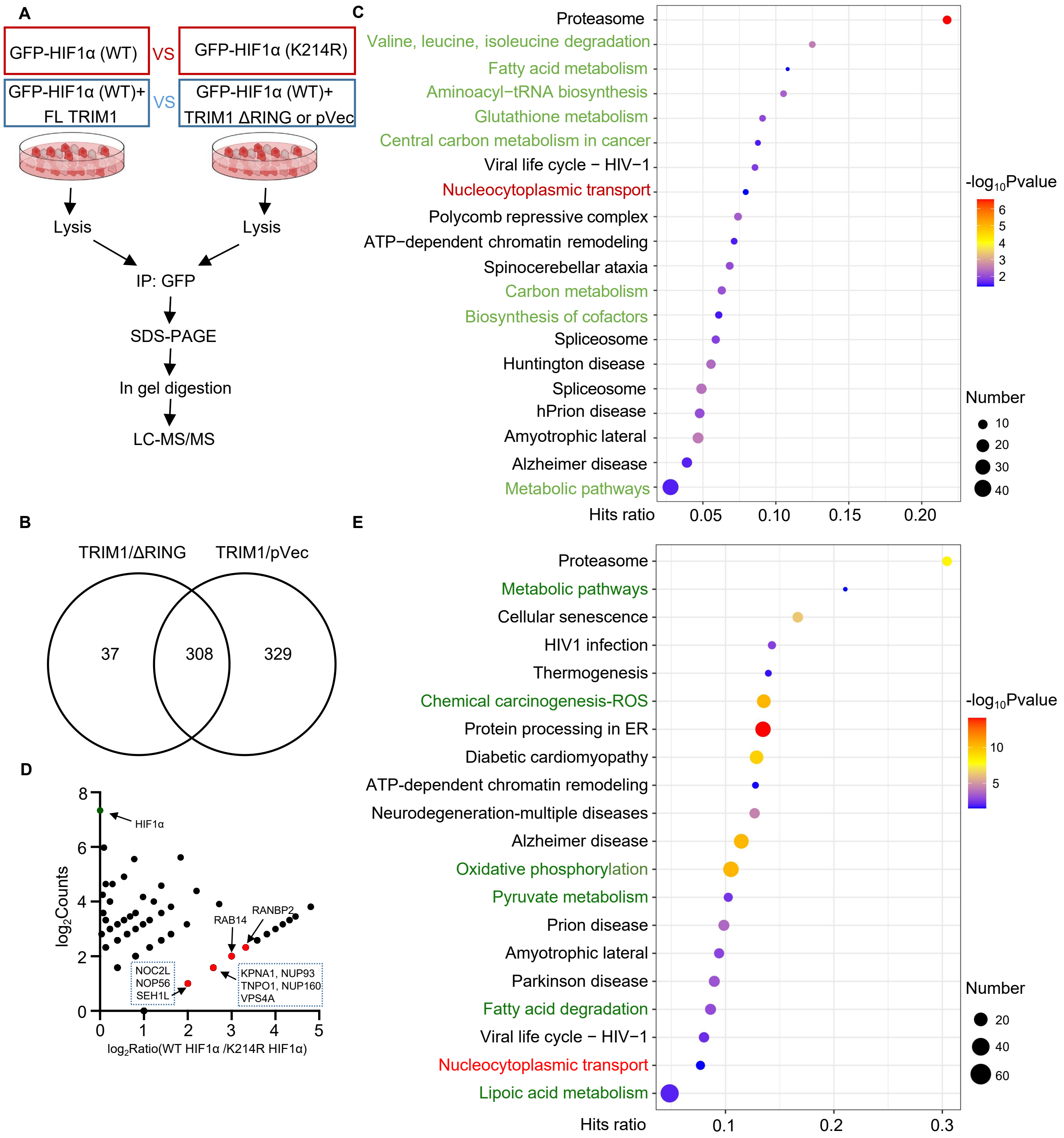

Supplementary Fig. S5: Quantification of differential HIF1α-binding proteins (HBPs) in SW480 cells by mass spectrometry analyses.

A Schematic diagram of the overall workflow that identifies potential HBPs. Lysates of cells transfected to express the indicated protein/combinations were subjected to IP with GFP-specific antibody. The precipitates were further separated by SDS-PAGE before in-gel digestion with trypsin and LC-MS/MS analyses.

B-C Quantification of the wild-type TRIM1-mediated HBPs in SW480 cells. GFP-HIF1α were co-transfected with Flag-tagged FL TRIM1, ΔRING or pVec. **B** Overlap of additional HBPs identified in FL TRIM1/ΔRING group and FL TRIM1/pVec group. **C** Pathway enrichment of the overlap HBPs in (B) by KEGG analyses using the DAVID online tool. The top 20 pathways were listed. The circle size represents the number of HBPs enriched in this pathway.

D-E Quantification of the HBPs in SW480 cells in wild type HIF1α/K214R HIF1α group. SW480 cells were transfected with GFP-HIF1α (WT) or GFP-HIF1α (K214R). **D** Scatter plots of protein ratios as a function of their relative abundance (denoted by MS/MS spectral counts). The ratio is calculated as spectral counts in GFP-HIF1α (WT) transfected samples divided by those in GFP-HIF1α (K214R). Higher ratios indicate increased binding efficiency with HIF1α. Red dots correspond to the potential HBPs involved in nuclear import, and the green dot corresponds to immunoprecipitated HIF1α. **E** Pathway enrichment of the HBPs in **(D)** by KEGG analyses using the DAVID online tool. The top 20 pathways were listed. The circle size represents the number of HBPs enriched in this pathway. This figure corresponds to Figure 9I.

Supplementary Table S1. The modification ratios of ubiquinated peptides from HIF1α

Peptide sequence	Peptide intensity				%Modification[3]	
	Modified[1]	Unmodifed[1]	Modified[2]	Unmodifed[2]	pVec	TRIM1
^{214}KPPmTcLVLIcEPIPHPSNIEIPLDSK^{240}	3561588	5567218	1442981	410088	39.0148	77.86979
^{33}ESEVFYELAHQLPLPHNVSSHLDK^{56}	3050897	12684465	2511843	6152112	19.3888	28.99187
^{274}SIYEYYHALDSDHLTK^{289}	4607929	44831650	6667539	35130403	9.32032	15.95184
^{637}ILIASPSPTHIHk^{649}	6729970	45262233	4451704	37517570	12.9442	10.60706
^{464}SSADPALNQEVALK^{477}	7068436	127943216	3905643	74568127	5.23543	4.977004
^{378}VESEDTSSLFDK^{389}	5630344	109613030	3029589	63703853	4.88561	4.539836
^{298}GQVTTGQYR^{306} (control)	-	1488430	-	1465585	-	-

1: Intensity of unmodified peptides in the HIF1α samples prepared from cells expressing pVec.
2: Intensity of unmodified peptides in the HIF1α samples prepared from cells expressing TRIM1.
3: Percentages are calculated as the ratio of the intensity of modified peptides in samples divided by that of total (unmodified plus the modified) peptides in the HIF1α samples.

Supplementary Table S2. qPCR primers used in this study

Gene	Primer sequence (5'-3')
ARNT2 Forward	GGAATGCCTACTCCAGTCTTGC
ARNT2 Reverse	CTTTGCCACTGCGACCAGACTT
PGK1 Forward	CCGCTTTCATGTGGAGGAAGAAG
PGK1 Reverse	CTCTGTGAGCAGTGCCAAAAGC
LDHA Forward	GGATCTCCAACATGGCAGCCTT
LDHA Reverse	AGACGGCTTTCTCCCTCTTGCT
TNFAIP3 Forward	CTCAACTGGTGTCGAGAAGTCC
TNFAIP3 Reverse	TTCCTTGAGCGTGCTGAACAGC
CCL5 Forward	CCTGCTGCTTTGCCTACATTGC
CCL5 Reverse	ACACACTTGGCGGTTCTTTCGG
RELB Forward	TGTGGTGAGGATCTGCTTCCAG
RELB Reverse	TCGGCAAATCCGCAGCTCTGAT
HIF1α Forward	TATGAGCCAGAAGAACTTTTAGGC
HIF1α Reverse	CACCTCTTTTGGCAAGCATCCTG
TRIM1 Forward	GCCAGTGTCTTGAACGGTCAAC
TRIM1 Reverse	GAGAAGATGCAGTTGCCATAGCG
GAPDH Forward	GTCTCCTCTGACTTCAACAGCG
GAPDH Reverse	ACCACCCTGTTGCTGTAGCCAA
β-actin Forward	CACCATTGGCAATGAGCGGTTC
β-actin Reverse	AGGTCTTTGCGGATGTCCACGT
